# Cannabinoids and opioid consumption in cancer pain: a systematic review and meta-analysis

**DOI:** 10.1007/s00520-026-10856-y

**Published:** 2026-06-09

**Authors:** Ioana Creangă-Murariu, Teodora Alexa-Stratulat, Matei Ioan Rusu, Stefan Chiru, Camelia Dascalu, Cristina-Elena Dobre, Bogdan-Ionel Tamba

**Affiliations:** 1 Advanced Center for Research and Development in Experimental Medicine “Prof. Ostin C. Mungiu”, Grigore T. Popa University of Medicine and Pharmacy, 16 Universitatii Street, 700115 Iasi, Romania; 2 Medical Oncology-Radiotherapy Department, Grigore T, Popa University of Medicine and Pharmacy, 16 University Street, 700115 Iasi, Romania; 3 Centre for Translational Medicine, Semmelweis University, 20 Baross Street, 1085 Budapest, Hungary; 4 Department of Medical Oncology, Regional Institute of Oncology, G-Ral M. Berthelot 9-11, 700483 Iasi, Romania; 5 Pharmacology and Algesiology, Grigore T. Popa University of Medicine and Pharmacy, 16 Universitatii Street, 700115 Iasi, Romania; 6 Socola Institute of Psychiatry, 36 Bucium Street, 700282 Iasi, Romania

**Keywords:** Cancer pain, Cannabinoids, Opioid consumption, Opioid-sparing, Systematic review, Meta-analysis

## Abstract

**Background:**

Opioids are the mainstay of cancer-related pain management but are limited by adverse effects and clinical complexity. Cannabinoids have been proposed as adjunctive, opioid-sparing agents, yet their impact on opioid consumption in cancer patients remains uncertain.

**Methods:**

This systematic review and meta-analysis was conducted according to PRISMA 2020 guidelines and registered in PROSPERO (CRD420251175971). Randomized and nonrandomized clinical studies involving adult cancer patients receiving opioids for pain and treated with cannabinoids were included. Outcomes comprised total opioid consumption, maintenance/background opioid dose, and breakthrough/rescue opioid use. Placebo-controlled comparisons were analyzed separately from within-group baseline changes. Risk of bias was assessed using RoB 2 and ROBINS-I, and certainty of evidence using GRADE.

**Results:**

Fifteen studies met inclusion criteria, with ten eligible for meta-analysis. Placebo-controlled analyses showed no significant differences between cannabinoids and placebo for total, maintenance, or breakthrough opioid use. Baseline-change analyses demonstrated heterogeneous and formulation-dependent effects, with modest reductions in maintenance opioid dose observed primarily in THC-predominant regimens, driven by isolated studies. Overall certainty of evidence was low due to heterogeneity and methodological limitations.

**Conclusions:**

Cannabinoids are not associated with consistent or clinically meaningful opioid-sparing effects in cancer pain under controlled conditions. Observed benefits in uncontrolled analyses are variable and not reliably reproduced. Cannabinoids should not be considered a dependable opioid-sparing strategy in cancer pain management.

**Supplementary Information:**

The online version contains supplementary material available at 10.1007/s00520-026-10856-y.

## Introduction

Cancer-related pain remains one of the most prevalent and distressing symptoms affecting patients, particularly in advanced stages of disease. Despite progress in oncologic treatments and supportive care, moderate-to-severe pain continues to be reported by approximately 40–60% of patients undergoing active cancer treatment and by more than 70% of those with advanced or metastatic disease [1]. Effective pain control is therefore a central component of comprehensive cancer care and a key determinant of quality of life.

Opioids remain the cornerstone of management for moderate to severe cancer-related pain and are recommended by international guidelines as first-line therapy when non-opioid measures are insufficient [2]. However, long-term opioid therapy is associated with substantial clinical challenges, including tolerance, opioid-induced hyperalgesia, constipation, nausea, sedation, endocrine dysfunction, cognitive impairment, and risk of dependence or misuse [3]. In patients with cancer, these adverse effects may exacerbate frailty, impair functional status, and complicate the delivery of anticancer therapies, leading to the so called “opioid use disorder” [4]. Consequently, there is a persistent clinical need for opioid-sparing strategies that can enhance analgesia while minimizing opioid exposure.

Cannabinoids have emerged as potential adjuvant agents in pain management due to their interaction with the endocannabinoid system, which plays a role in nociceptive modulation, inflammation, and affective components of pain [5]. Cannabinoid receptors (CB1R and CB2R) are expressed either throughout the central and peripheral nervous systems or in immune cells, and their activation has been shown to modulate neurotransmitter release and nociception signaling pathways relevant to pain processing [6]. Importantly, preclinical studies suggest functional interactions between cannabinoid and opioid systems, including receptor cross-talk and convergent intracellular signaling, raising the possibility that cannabinoids could interfere with opioid effects [7]

Although the preclinical research is abundant on positive effects of cannabinoids as adjuvants for opioids, early randomized trials suggested modest analgesic benefit of THC:CBD oromucosal spray in opioid-refractory cancer pain, but results were inconsistent and opioid dose reduction was rarely assessed as a primary outcome [8]. Evidence syntheses mirror these findings. Cancer-pain-specific reviews report low-certainty analgesic benefit with frequent adverse effects, particularly for THC-predominant products [9], while broader opioid-sparing reviews indicate that observational signals of opioid reduction are not consistently supported by randomized evidence [10]. Importantly, opioid outcomes have been inconsistently defined, often relying on uncontrolled pre-post comparisons without distinguishing placebo-controlled effects across opioid domains.

Given the central role of opioids in the management of cancer-related pain and the increasing clinical and patient-driven interest in cannabinoids as adjunctive treatments, a focused and methodologically rigorous synthesis examining opioid consumption outcomes in cancer patients is needed. Distinguishing whether cannabinoids are associated with true reductions in opioid requirements, rather than apparent changes arising from non-specific or uncontrolled influences, is critical for informing clinical practice, guiding evidence-based recommendations, and shaping future trial design.

Although previous reviews have reported opioid-related outcomes in cannabinoid studies, the specific question of opioid-sparing in opioid-treated cancer patients remains insufficiently resolved [9, 10]. This issue should be treated differently than opioid reduction in broader chronic pain populations, because opioid use in cancer is influenced by disease progression, rescue medication policies, and palliative treatment goals. The present review addresses this gap through an updated cancer-specific synthesis that includes both randomized and observational evidence, incorporates recent evidence, and separates controlled treatment effects from within-group baseline changes across total opioid consumption, maintenance/background dose, and breakthrough opioid use.

Accordingly, the aim of this systematic review and meta-analysis is to evaluate the opioid-sparing effects of cannabinoids in adult cancer patients with persistent pain receiving opioid analgesics, by quantitatively synthesizing available evidence on opioid use.

## Methods

### Reporting standards and protocol registration

This systematic review and meta-analysis was performed and reported in accordance with the PRISMA 2020 statement (Supplementary Table 1 (S)) [11] and followed the methodological guidance outlined in the Cochrane Handbook for Systematic Reviews of Interventions [12]. The review was prospectively registered in the PROSPERO database (registration number CRD420251175971).

### Eligibility criteria

We considered clinical studies involving adult patients (≥ 18 years) diagnosed with cancer and experiencing pain of any etiology (tumor-related, treatment-induced, or mixed) while receiving opioid analgesic therapy. Eligible interventions included any cannabinoid-based treatment administered alone or as an adjunct to opioids, encompassing phytocannabinoids (such as THC or CBD), synthetic cannabinoids (including dronabinol and nabilone), and combined or standardized cannabinoid formulations (e.g., nabiximols), irrespective of dose, route of administration, or treatment duration.

Eligible comparators included placebo or standard care without cannabinoids, as well as within-group baseline values in studies reporting pre-post changes in opioid consumption. Both randomized controlled trials and nonrandomized clinical studies (interventional or observational) were included. No restrictions were applied regarding language or year of publication. To be eligible, studies were required to report quantitative measures of opioid consumption, such as daily opioid dose (e.g., morphine milligram equivalents), cumulative opioid exposure, maintenance/background opioid dose, or breakthrough/rescue opioid use. Studies lacking extractable opioid consumption data were excluded.

### Information sources

A comprehensive literature search was conducted on 24th October 2025 in PubMed, Embase (via Ovid), and the Cochrane Central Register of Controlled Trials (CENTRAL), covering all records from database inception to the most recent search update prior to data synthesis. No limits were imposed on language or publication date. In addition, manual reference list screening of included studies and relevant reviews was undertaken to identify additional eligible publications using *citationchaser* (Version 2.0, Stockholm Environment Institute, Sweden) [13]. When necessary, corresponding authors were contacted to clarify reported outcomes or to obtain missing numerical data. The detailed search key can be found in supplementary, Table 2 (S).

### Selection process

Following duplicate removal using *Endnote 20* (Clarivate, 2013), all identified records underwent independent title and abstract screening by at least two reviewers (IC-M, SC) using *Rayyan* (Version 1.0, Qatar Computing Research Institute (QCRI), Qatar) [14]. Articles deemed potentially eligible were retrieved in full and assessed independently against the predefined inclusion criteria. Cohen’s kappa coefficient (k) was calculated to measure inter-rate reliability after each selection step. Any disagreements during the screening or eligibility assessment process were resolved through discussion, with involvement of a senior reviewer (TA-S), when consensus could not be reached.

### Data collection process

Data extraction was performed independently by two reviewers (IC-M, SC) using a standardized extraction template. Extracted variables included study design, sample size, characteristics of the cancer population, details of the cannabinoid intervention (compound, formulation, dose, and duration), comparator characteristics, opioid treatment context, and quantitative opioid consumption outcomes. Cannabinoid interventions were categorized into THC-predominant, CBD-predominant, or balanced THC:CBD combination groups where sufficient information was available. When outcome data required for meta-analysis were incomplete or unclear, attempts were made to obtain additional information directly from study authors.

### Study risk of bias assessment and certainty of evidence

The methodological quality of included studies was evaluated independently by at least two reviewers. Randomized controlled trials were assessed using the Cochrane Risk of Bias 2 (RoB 2) tool [15], while nonrandomized studies were evaluated with the ROBINS-I instrument [16]. Disagreements in risk-of-bias judgments were resolved through discussion.

The certainty of evidence for each pooled clinical outcome was evaluated using the GRADE (Grading of Recommendations Assessment, Development and Evaluation) approach, and the GRADEpro tool (Version 3.2, McMaster University, Canada), considering risk of bias, inconsistency, indirectness, imprecision, and potential publication bias [17].

### Synthesis methods

Where appropriate, data were pooled using random-effects meta-analysis, reflecting anticipated clinical and methodological heterogeneity across studies. For continuous opioid consumption outcomes, mean differences (MD) were calculated when outcomes were reported using the same measurement units, whereas standardized mean differences (SMD) were applied when different scales or units were used.

Clinical outcomes were synthesized separately based on the type of comparison reported, distinguishing between-group placebo-controlled analyses from within-group change-from-baseline analyses, which were analyzed independently to avoid conflating controlled treatment effects with uncontrolled temporal changes. Prespecified subgroup analyses were conducted according to cannabinoid formulation (THC-predominant, CBD-predominant, or balanced THC:CBD combinations) and opioid outcome domain (total opioid consumption, maintenance/background opioid dose, and breakthrough/rescue opioid use). Statistical heterogeneity was quantified using the I^2^ statistic, and subgroup differences were examined using standard interaction tests [18]. When quantitative synthesis was not feasible, findings were summarized using a structured narrative approach. All statistical analyses were calculated by R software using the meta5 package for basic meta-analysis calculations and plots, and the dmetar6 package for additional influential analysis calculations and plots.

## Results

### Characteristics of included studies

**Fig. 1:**
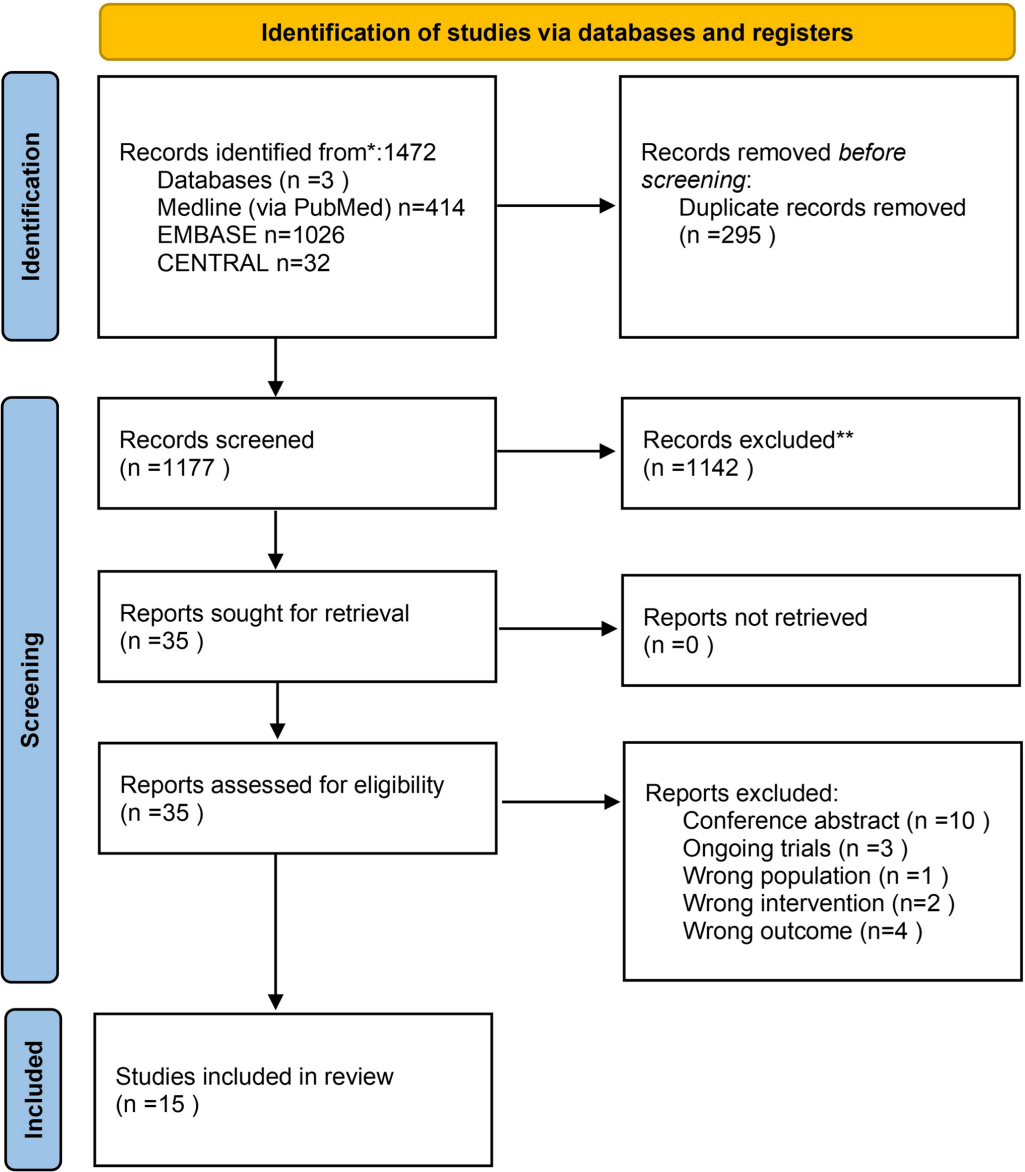
PRISMA flowchart of included articles

Fifteen articles met the inclusion criteria, however only ten were eligible for meta-analysis, due to non-poolable opioid consumption data, incomplete reporting, lack of variance measures, or outcome definitions that were not comparable across studies. These details are presented in Fig. 1. Among them, nine articles were RCTs [19–27], while six were observational [28–33] Patients received either THC-predominant drugs [19, 27, 29], CBD-predominant [23, 27], or more often drugs with balanced THC:CBD formulations [19, 20, 22, 24–26, 28, 30–34]. Most often the drug was delivered in an oromucosal spray [19–21, 25, 26, 30] or oral oil [22, 23, 27], but others included all forms such as oral, inhalatory or topical [24, 28, 29, 31–33]. Baseline characteristics of included studies is exposed in Table 1.

**Table 1:** Baseline characteristics table

Author	Study duration (days)	No of centers involved	No of patients enrolled (intervention/control)	Age (mean ± SD)	Previous cannabinoid exposure (intervention/control)
Johnson et al. 2010	16	10	118/118	59.40 ± 12.10	6/7
Portenoy et al. 2012	63	9	269/273	59 ± 12.3	11/6
Clarke et al. 2022	30	1	25	55.9 ± 11.9	0
Fallon et al. 2017	35	101	200/199	60 ± 11	4/0
Good et al. 2020	14	1	21/18	57.7 ± 12.4	0/0
Hardy et al. 2022	28	5	70/72	63.6 ± 14	0/0
Zylla et al. 2021	84	1	15/15	57 ± 9	0/0
Lichtman et al. 2018	35	114	199/198	59.2 ± 12	0/0
Pitchard et al. 2019	84	1	22/61	48 ± 12.16	0/0
Aprikian et al. 2023	21	1	197	57.6 ± 14.7	0
Hardy et al. 2025	28	5	72/72	65 ± 3.6	0/0
Bramness et al. 2021	10	N/A	607	52.9 ± 13.5	0
Pawasarat et al. 2020	50	1	137/95	57 ± 14	0/0
Sura et al. 2022	30	1	184	59.8 ± 12.73	13
Webster et al. 2020	10	1	31	63 ± 2.4	31

### Total opioid consumption

**Fig. 2:**
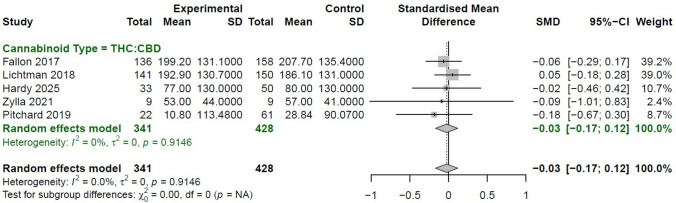
Effect of cannabinoids versus placebo on daily total opioid consumption. Forest plot showing the pooled mean difference in daily total opioid dose between cannabinoid and placebo groups, stratified by cannabinoid type, with no significant overall opioid-sparing effect observed under placebo-controlled conditions. Abbreviations: CI, confidence interval; MD, mean difference; SE, standard error; THC, Δ9-tetrahydrocannabinol; CBD, cannabidiol

In placebo-controlled analyses, total daily opioid consumption did not differ significantly between cannabinoid-treated and placebo-treated participants. Five randomized controlled trials evaluating THC:CBD formulations [22, 24, 25, 28, 34], encompassing a total of 769 participants, were included. The pooled SMD was −0.03 (95% CI −0.17; 0.12), indicating no measurable reduction in opioid use associated with cannabinoid therapy, as exposed in Fig. 2.

**Fig. 3:**
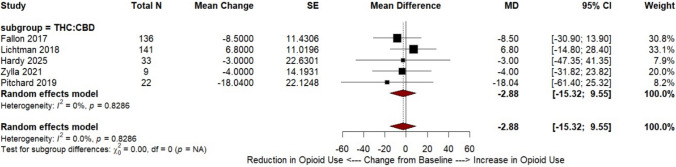
Change in daily total opioid consumption from baseline. Forest plot summarizing within-group changes in daily total opioid use from baseline following cannabinoid treatment, indicating heterogeneous and non-significant overall effects. Abbreviations: CI, confidence interval; MD, mean difference; SE, standard error; THC, Δ9-tetrahydrocannabinol; CBD, cannabidiol

When total opioid consumption was assessed as change from baseline within cannabinoid-treated groups, a numerical reduction was observed, although this effect did not reach statistical significance. The pooled MD was −2.88 (95% CI −15.32; 9.55). Individual studies demonstrated variable baseline changes, ranging from modest reductions to slight increases in opioid use, and all confidence intervals overlapped zero. Results are presented in Fig. 3.

### Maintenance opioid dose

**Fig. 4:**
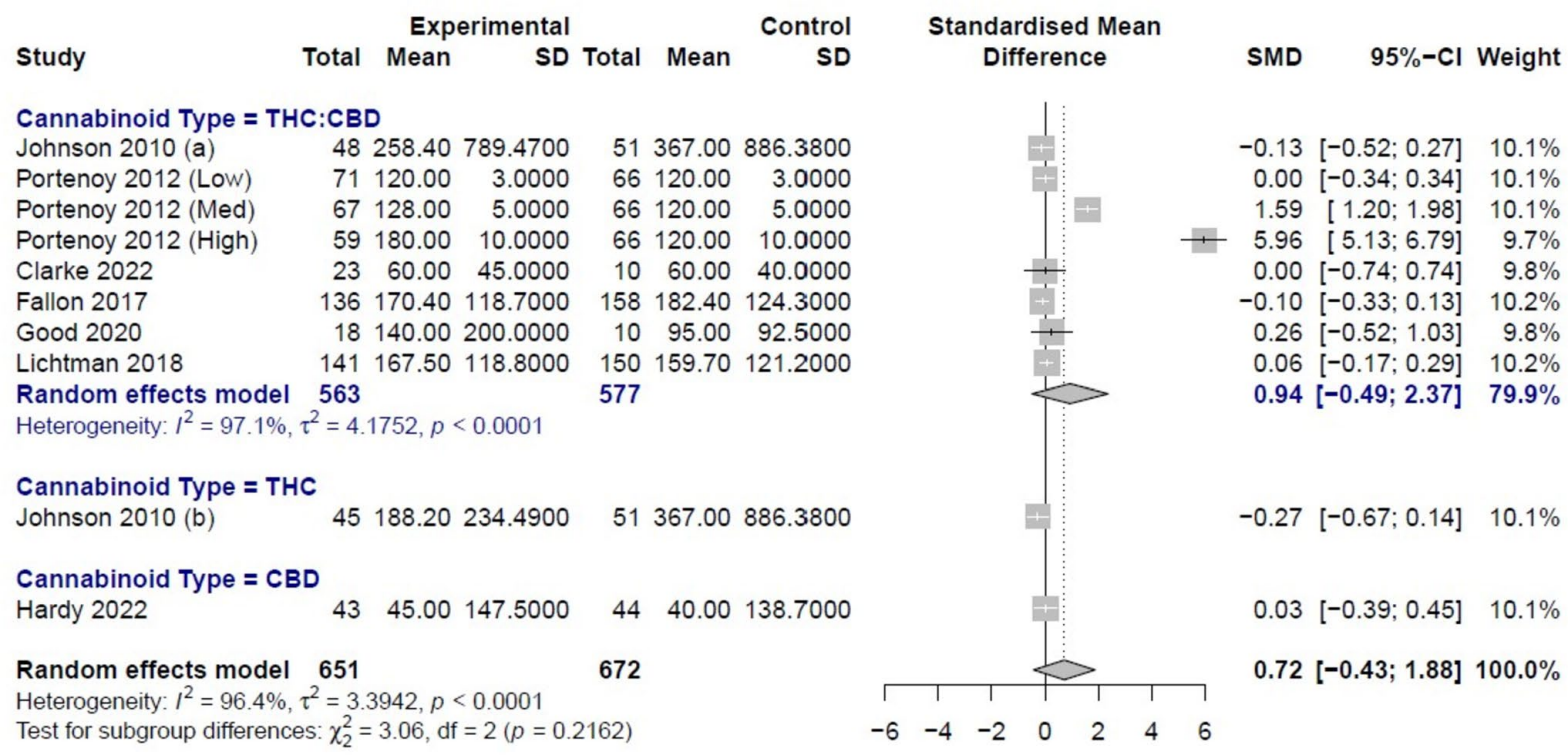
Effect of cannabinoids versus placebo on maintenance opioid dose. Forest plot presenting standardized mean differences in maintenance opioid dose between cannabinoid and placebo groups, demonstrating no meaningful between-group differences. Abbreviations: CI, confidence interval; SMD, standardized mean difference; SD, standard deviation; THC, Δ9-tetrahydrocannabinol; CBD, cannabidiol

Placebo-controlled analyses of maintenance opioid dosing demonstrated substantial variability and no consistent evidence of opioid-sparing effects associated with cannabinoid use (Fig. 4). Seven randomized comparisons, stratified by cannabinoid formulation, were included [19, 20, 23, 25–27, 34]. The overall pooled effect was not clinically relevant, nor statistically significant (MD 0.72 (95% CI −0.43; 1.88) and was characterized by high heterogeneity (I^2^ = 96.4%, p < 0.0001), reflecting marked between-study variability. Subgroup analysis by cannabinoid type did not reveal statistically significant differences between balanced THC:CBD combinations, THC-predominant regimens, or CBD-predominant formulations (p = 0.216).

**Fig. 5:**
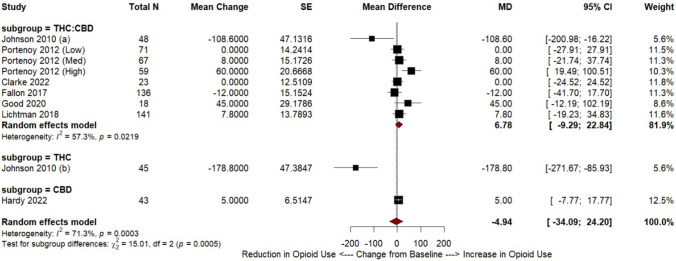
Change in maintenance opioid dose from baseline. Forest plot showing changes in maintenance opioid requirements from baseline after cannabinoid administration, with pooled estimates showing no significant opioid reduction. Abbreviations: CI, confidence interval; MD, mean difference; SE, standard error; THC, Δ9-tetrahydrocannabinol; CBD, cannabidiol

In contrast, baseline-change analyses of maintenance opioid dose revealed marked heterogeneity and statistically significant differences between cannabinoid subgroups (Fig. 5). Across all formulations, the pooled MD was −4.94 mg (95% CI −34.09; 24.20), with substantial heterogeneity (I^2^ = 71.3%). Importantly, the test for subgroup differences was statistically significant (p = 0.0005). THC-only interventions demonstrated pronounced baseline reductions in maintenance opioid dosing, driven predominantly by Johnson et al. trial [19] which reported a mean reduction of −178.8 (95% CI −271.7; −85.9). In contrast, THC:CBD combinations exhibited heterogeneous effects, with individual studies reporting both increases and decreases in opioid dose, resulting in a non-significant pooled estimate. CBD-only regimens showed minimal change from baseline, with a pooled MD of 5.0 (95% CI −7.77; 17.77).

### Breakthrough opioid consumption

**Fig. 6:**
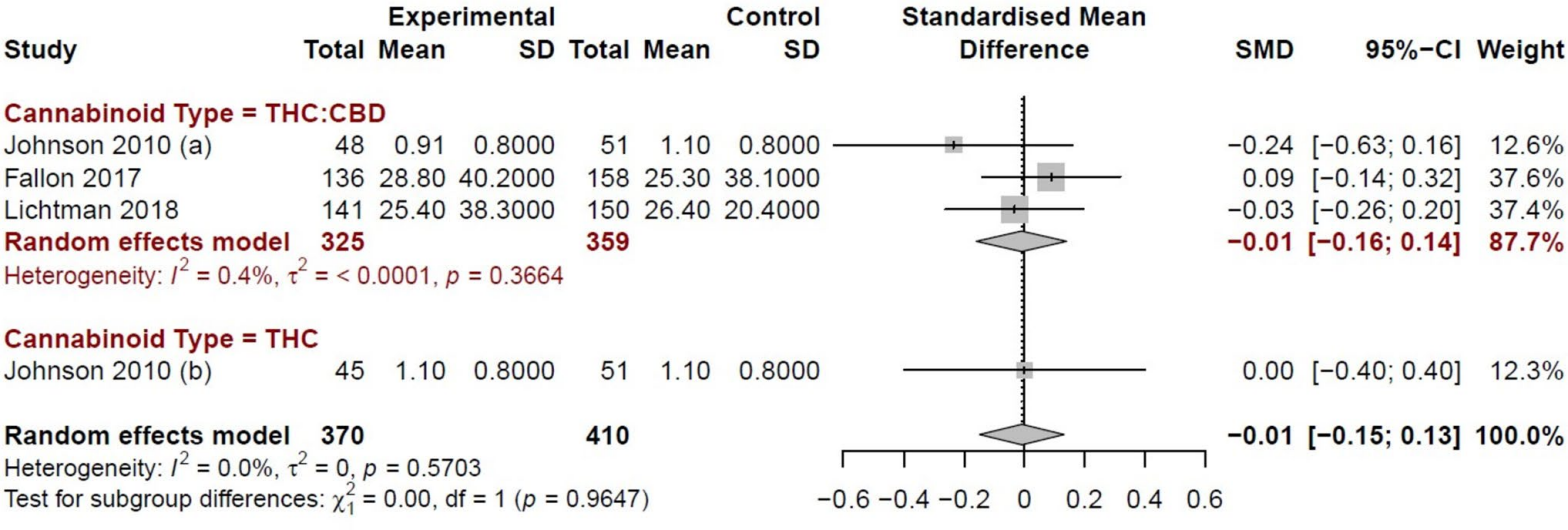
Effect of cannabinoids versus placebo on breakthrough opioid use. Forest plot illustrates the effect of cannabinoids on breakthrough opioid consumption compared with placebo, indicating no significant opioid-sparing benefit. Abbreviations: CI, confidence interval; SMD, standardized mean difference; SD, standard deviation; THC, Δ9-tetrahydrocannabinol; CBD, cannabidiol

In placebo-controlled analyses, cannabinoid therapy was not associated with significant changes in breakthrough (rescue) opioid use. Three randomized trials assessing THC:CBD and THC-only formulations were included [19, 25, 34]. The pooled SMD ranged from −0.01 to −0.03, with all confidence intervals crossing zero. Between-study heterogeneity was minimal, and individual trial estimates demonstrated near-identical rescue opioid requirements in cannabinoid and placebo groups. No statistically significant subgroup differences by cannabinoid formulation were observed. Results are presented in Fig. 6.

**Fig. 7:**
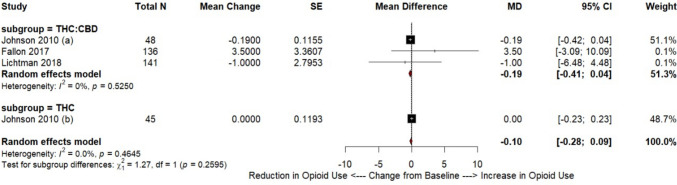
Change in breakthrough opioid use from baseline. Forest plot showing within-group changes in breakthrough opioid use following cannabinoid treatment, with pooled results demonstrating no statistically significant reduction. Abbreviations: CI, confidence interval; MD, mean difference; SE, standard error; THC, Δ9-tetrahydrocannabinol; CBD, cannabidiol

Baseline-anchored analyses similarly demonstrated no meaningful changes in breakthrough opioid use following cannabinoid administration (Fig. 7). For balanced THC:CBD formulations, the pooled MD was −0.19 (95% CI −0.41; 0.04), while THC-predominant regimens yielded a pooled MD of 0.00 (95% CI −0.23; 0.23). Across studies, rescue opioid use remained stable relative to baseline, indicating no detectable impact of cannabinoid therapy on episodic opioid requirements.

### Risk of bias and certainty assessment

**Fig. 8:**
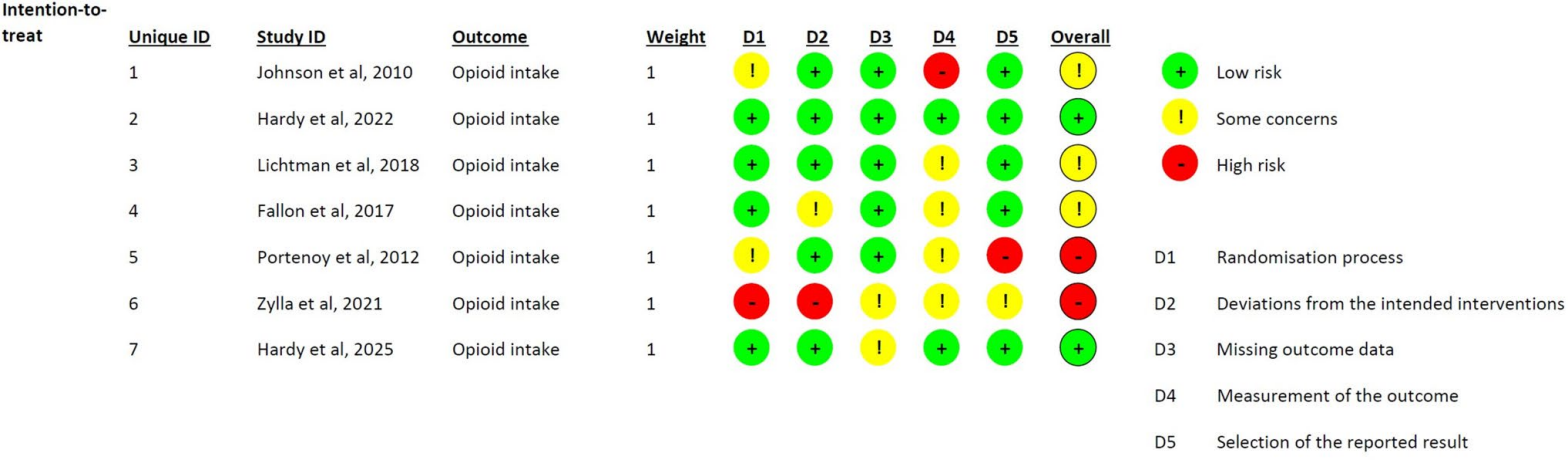
Risk of bias assessment of randomized controlled trials (RoB 2). Summary of the risk of bias across five RoB 2 domains for randomized controlled trials evaluating cannabinoid effects on opioid intake, with most studies rated as low risk or with some concerns. Abbreviations: RoB 2, Risk of Bias 2 tool; D1, randomization process; D2, deviations from intended interventions; D3, missing outcome data; D4, measurement of the outcome; D5, selection of the reported result

Across included trials, risk of bias of RCTs was generally low, with most domains showing adequate randomization, blinding, and outcome measurement. The main source of uncertainty was missing outcome data, reflecting attrition common in advanced cancer populations, while selective reporting was unlikely. Figure 8 presents the details.

**Fig. 9:**
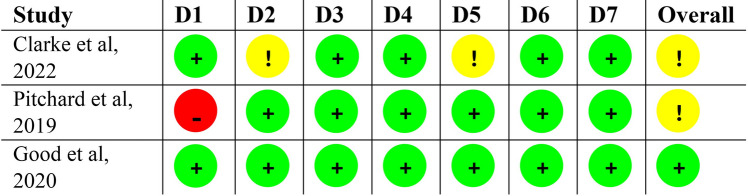
Risk of bias assessment of nonrandomized studies (ROBINS-I). Risk of bias across ROBINS-I domains for nonrandomized studies assessing cannabinoid-opioid interactions, highlighting variability in confounding and selection bias. Abbreviations: ROBINS-I, Risk Of Bias In Nonrandomized Studies of Interventions; D1, confounding; D2, selection of participants; D3, classification of interventions; D4, deviations from intended interventions; D5, missing data; D6, measurement of outcomes; D7, selection of the reported result

Nonrandomized studies demonstrated variable risk of bias, with most domains rated as low or moderate risk. The primary sources of concern related to confounding and participant selection, reflecting the observational design and limited control over baseline opioid use and disease trajectory. In contrast, bias due to outcome measurement and reporting was generally low, suggesting that observed uncertainties are driven mainly by study design rather than selective reporting or outcome assessment, as exposed in Fig. 9.

The certainty of evidence assessed with the GRADE tool was evaluated as low for all outcomes, mainly due to population heterogeneity and lack of uniformity in intervention administration, as seen in Supplementary Tables 3 (S).

## Discussion

This meta-analysis evaluated whether adjunctive cannabinoids reduce opioid need of cancer patients while preserving optimal pain control, including analyses across total daily opioid dose, maintenance opioid dose, and breakthrough opioid use. Overall, cannabinoids were associated with limited benefits in cancer population, without consistent evidence of reduced opioid requirements. Across outcomes, effects on total, maintenance, and breakthrough opioid use were largely neutral, and findings were characterized by substantial variability and inconsistency, indicating that cannabinoids do not provide a reliable opioid-sparing advantage in cancer pain management.

Overall opioid consumption and the need for breakthrough analgesia remained largely unchanged, indicating that cannabinoids do not substantially modify background opioid exposure or episodic pain requiring rescue medication. Although modest reductions in opioid use were observed in selected settings, most notably for maintenance dosing and primarily with THC-based interventions, these effects were variable, formulation dependent, and not consistently reproduced across studies. Taken together, the findings suggest that any apparent opioid-sparing effects of cannabinoids are limited, heterogeneous, and context dependent, and do not support a robust or generalizable reduction in opioid use in cancer pain management.

However, opioid-sparing effect remains mechanistically plausible because endocannabinoid signaling intersects with central nociceptive modulation and may influence affective pain processing, sleep disruption, anxiety, and symptom appraisal [35]. However, cancer pain in opioid-treated populations is not a static phenotype: pain intensity and opioid requirements fluctuate with tumor progression, inflammatory burden, anticancer therapy, infections, and functional decline [36]. In advanced cancer, clinical priorities commonly emphasize comfort and stability over opioid minimization, and opioid titration is constrained by clinical urgency, adverse effects, and care setting [21]. Consequently, even if cannabinoids provide modest symptom benefit, such improvements may not translate into intentional opioid tapering, particularly within the short follow-up windows typical of cannabinoid RCTs. This clinical context plausibly contributes to the consistently null placebo-controlled effects for total dose and breakthrough opioid use, which integrate multiple downstream processes (opioid titration decisions, rescue policies, intercurrent therapies, and disease trajectory) that can dilute small treatment signals.

Importantly, several RCTs constrained the opioid dosing of the enrolled patients by protocol [19, 20, 25, 34]. Background opioid doses were commonly required to remain stable throughout follow-up, and breakthrough opioid use was restricted by predefined limits, with excessive rescue use sometimes leading to patient exclusion. Under these conditions, potential cannabinoid-related benefits may have been attenuated or masked in opioid-consumption outcomes, since reductions in opioid requirements could not always be captured by trial design. In consequence, the neutral opioid-consumption findings may reflect, at least in part, restricted opportunity for opioid dose modification rather than the complete absence of cannabinoid-related clinical benefit.

The divergence between placebo-controlled comparisons and change-from-baseline analyses is methodologically expected and clinically informative. Baseline-change designs are intrinsically susceptible to regression to the mean, especially when patients enroll during high-symptom periods; subsequent improvement may occur independently of the intervention [37]. Expectancy effects may further contribute, as cannabinoids are associated with strong patient beliefs regarding analgesia and opioid substitution, potentially influencing self-management behaviors and symptom reporting; however, such effects are attenuated in blinded placebo-controlled trials [38]. In addition, opioid titration rules vary across studies, and permissive titration can generate large within-person opioid changes that are not attributable to treatment, inflating heterogeneity in baseline analyses and potentially contributing to the apparent THC-predominant signal in maintenance dosing. Finally, dose-limiting adverse effects may cap cannabinoid exposure, preventing consistent pharmacologic effects across trials and yielding small, inconsistent signals that do not persist under placebo control.

Cannabinoids are often associated with benefits for symptom palliation beyond analgesia. A recent systematic review and meta-analysis conducted by our group synthesized a broad evidence base and reported that cannabinoids were associated with improvements in pain and anxiety compared with baseline values [8]. Importantly, this review also emphasized safety trade-offs, particularly with THC-predominant formulations, reporting increased risks of psychiatric and neurological adverse effects [8]. These harms are clinically silent in opioid-treated cancer patients, who frequently have baseline vulnerability to sedation, cognitive impairment, delirium, falls, and polypharmacy interactions [39]. Moreover, tolerability constraints may limit cannabinoid dose escalation and thereby constrain any potential analgesic or opioid-sparing effects in practice. These opioid-focused findings help contextualize symptom-level results, indicating that while cannabinoids may be associated with within-patient improvements in pain or anxiety in uncontrolled analyses, such improvements do not consistently translate into reductions in opioid use under blinded conditions and may be limited by tolerability and adverse effects.

Clinically, opioid-sparing should not be pursued as an isolated objective in cancer pain management. In advanced cancer and end-of-life care, adequate symptom relief and quality of life remain the primary goals, and appropriately prescribed opioids continue to have a central role [40, 41]. Therefore, adjunctive cannabinoids should not be introduced solely to reduce opioid dose, particularly given their own adverse-effect profile, as previously mentioned. Any opioid-sparing strategy should be considered clinically meaningful only if it reduces opioid exposure without worsening pain control, function, quality of life, or safety.

Our study’s findings are broadly aligned with prior systematic reviews and meta-analyses evaluating cannabinoids in cancer-related pain, particularly in underscoring that effects observed in uncontrolled or baseline-based analyses are often attenuated in placebo-controlled settings. Reviews focused specifically on cancer pain and have generally reported limited or uncertain analgesic benefit when cannabinoids are added to opioid therapy in rigorously controlled trials, suggesting that meaningful downstream reductions in opioid use are unlikely. In this context, the Cochrane review of cannabis-based medicines for adults with cancer pain emphasized both the low certainty of efficacy and the relevance of adverse effects, a pattern that is consistent with the absence of robust opioid-sparing effects observed across total, maintenance, and breakthrough opioid outcomes in controlled analyses [9].

Similar discrepancies between uncontrolled and randomized evidence have been described in broader chronic pain populations receiving prescribed opioids, where observational studies more frequently report reductions in opioid use, while randomized trials yield smaller, inconsistent, or null effects and are limited by substantial confounding. The article conducted by Noori et al.reflects a comparable structure within opioid-treated cancer pain, in which modest baseline changes, most apparent for maintenance dosing and largely confined to THC-based interventions, are not consistently reproduced in controlled comparisons [10]. The same trend was emphasized by Nielsen et al.where the analysis found no consistent evidence of opioid reduction in randomized controlled trials, despite more favorable signals in uncontrolled or observational data [42]

A key strength of this review is its focused assessment of opioid-sparing in opioid-treated cancer patients, distinguishing it from broader reviews of cannabinoids for cancer pain or chronic pain. We separately analyzed total opioid consumption, maintenance/background opioid dose, and breakthrough/rescue opioid use, rather than treating opioid use as a single outcome. We also distinguished placebo-controlled effects from within-group baseline changes, improving causal interpretation while contextualizing opioid trajectories over time. Also, subgrouping by cannabinoid formulation increased clinical interpretability given expected differences in efficacy and tolerability across THC-predominant, balanced THC:CBD, and CBD-predominant products.

The main limitations of this review reflect the nature of the available evidence, which is both heterogeneous and only partly aligned with the clinical question of opioid-sparing. From a clinical perspective, opioid-sparing requires more than a numerical reduction in opioid dose; it implies a sustained decrease in opioid exposure without deterioration in pain control, function, quality of life, or safety [43]. Most available studies were not designed around this composite clinical objective; instead, opioid consumption was often captured as a secondary or exploratory measure within trials primarily evaluating analgesic efficacy, symptom control, or tolerability.

This creates several interpretive challenges. First, opioid outcomes are highly dependent on protocol conditions, including baseline opioid optimization, allowing dose adjustments, rescue medication rules, tapering procedures, and follow-up duration. If opioid dosing is kept stable or rescue use is capped, a potential symptomatic benefit may not translate into measurable opioid reduction. Conversely, in uncontrolled studies, reductions in opioid use may reflect disease trajectory, regression to the mean, patient preference, clinician behavior, or expectancy effects rather than a true pharmacologic opioid-sparing effect. Second, the included populations vary widely in cancer type, disease stage, prognosis, baseline opioid exposure, and palliative care context, all of which influence whether opioid reduction is clinically appropriate or feasible. Third, cannabinoid interventions differ substantially in formulation, THC:CBD ratio, dose, route of administration, titration schedule, and tolerability profile, limiting the ability to identify whether any opioid-sparing signal is formulation-specific or patient-specific.

Patient-level variability represents another important source of uncertainty in opioid-specific outcomes. Opioid requirements in cancer pain are influenced not only by pain intensity, but also by duration of prior opioid therapy, opioid tolerance, cancer diagnosis and stage, pain mechanism, performance status, renal and hepatic function, concomitant analgesics or sedating medications, and exposure to anticancer treatments [44]. In addition, inter-individual differences in opioid pharmacokinetics and pharmacodynamics, including genetic variability affecting opioid metabolism and receptor sensitivity, may contribute to substantial variation in the dose required to achieve adequate analgesia [45]. These factors may obscure cannabinoid-related effects if they are not measured, balanced, or adjusted for in trial analyses.

These gaps have direct implications for clinical practice: the available evidence does not allow clinicians to reliably identify which patients, if any, are likely to reduce opioid requirements with cannabinoids, nor under what dosing strategy this could be achieved safely. Future research should therefore move from simply recording opioid consumption toward prospectively testing opioid-sparing strategies. Trials should define opioid reduction as a primary or key secondary endpoint, use standardized morphine-equivalent reporting, distinguish maintenance from breakthrough opioid use, incorporate prespecified tapering or opioid-adjustment algorithms, and evaluate whether opioid reductions occur without worsening pain, function, sleep, psychological distress, or adverse-event burden. RCTs and high-quality observational studies may also be useful, provided they carefully control baseline opioid dose, disease progression, anticancer treatment, performance status, and palliative care involvement. Such designs would better determine whether cannabinoids have a clinically meaningful opioid-sparing role, rather than merely showing whether opioid use changes over time. Until such evidence is available, cannabinoids should be viewed as potential adjunctive symptom-directed therapies rather than established opioid-sparing interventions in cancer pain management.

## Conclusion

In this systematic review and meta-analysis, cannabinoids were not associated with consistent or clinically meaningful reductions in opioid use among cancer patients receiving opioid analgesics. While modest and context-specific signals of benefit were observed in selected settings, particularly for maintenance dosing, these effects were heterogeneous and not reliably reproduced in controlled analyses. Overall, the findings do not support cannabinoids as a dependable opioid-sparing strategy in cancer pain management, although a limited adjunctive role for symptom control in carefully selected patients cannot be excluded.

## Supplementary Information

Below is the link to the electronic supplementary material.

Supplementary file 1 (DOCX 27.6 KB)

## Data Availability

No datasets were generated or analyzed during the current study.

## References

[1] Snijders RAH, Brom L, Theunissen M, van den Beuken-van Everdingen MHJ (2023) Update on prevalence of pain in patients with cancer 2022: a systematic literature review and meta-analysis. Cancers (Basel) 15:591. 10.3390/CANCERS15030591/S1 36765547 10.3390/cancers15030591 PMC9913127

[2] Pasricha SV, Tadrous M, Khuu W, Juurlink DN, Mamdani MM, Michael Paterson J, Gomes T (2018) Clinical indications associated with opioid initiation for pain management in Ontario, Canada: a population-based cohort study. Pain 159:1562–1568. 10.1097/J.PAIN.0000000000001242 29762260 10.1097/j.pain.0000000000001242 PMC6085129

[3] Benyamin R, Trescot AM, Datta S, Buenaventura R, Adlaka R, Sehgal N, Glaser SE, Vallejo R (2008) Opioid complications and side effects. Pain Physician 11(2Suppl):S105–S120 18443635

[4] Lewis CES, Schutzer-Weissmann J, Farquhar-Smith P (2023) Opioid use disorder in cancer patients. 10.1097/SPC.0000000000000640 10.1097/SPC.0000000000000640 36866646

[5] Woodhams SG, Sagar DR, Burston JJ, Chapman V (2015) The role of the endocannabinoid system in pain. Handb Exp Pharmacol 227:119–143. 10.1007/978-3-662-46450-2_7 25846617 10.1007/978-3-662-46450-2_7

[6] Kendall DA, Yudowski GA (2017) Cannabinoid receptors in the central nervous system: their signaling and roles in disease. Front Cell Neurosci 10:294, 294. 10.3389/FNCEL.2016.00294 10.3389/fncel.2016.00294 PMC5209363 28101004

[7] Scavone JL, Sterling RC, Van Bockstaele EJ (2013) Cannabinoid and opioid interactions: implications for opiate dependence and withdrawal. Neuroscience 248:637. 10.1016/J.NEUROSCIENCE.2013.04.034 23624062 10.1016/j.neuroscience.2013.04.034 PMC3742578

[8] Creangă-Murariu I, Rezuș II, Karami R, Rancz A, Zolcsák Á, Engh MA, Obeidat M, Tamba BI, Hegyi P, Bunduc S (2025) Indications of cannabinoids for the palliation of cancer-associated symptoms: a systematic review and meta-analysis. Curr Oncol Rep 27:1080–1096. 10.1007/S11912-025-01695-X 40748522 10.1007/s11912-025-01695-x PMC12450232

[9] Häuser W, Welsch P, Radbruch L, Fisher E, Bell RF, Moore RA (2023) Cannabis-Based Medicines and Medical Cannabis for Adults with Cancer Pain. Cochrane Database Syst Rev 6. 10.1002/14651858.CD014915.PUB2 10.1002/14651858.CD014915.pub2 PMC10241005 37283486

[10] Noori A, Miroshnychenko A, Shergill Y, Ashoorion V, Rehman Y, Couban RJ, Buckley ND, Thabane L, Bhandari M, Guyatt Gea, Buckley DN et al (2021) Opioid-sparing effects of medical cannabis or cannabinoids for chronic pain: a systematic review and meta-analysis of randomised and observational studies. BMJ Open 11:e047717. 10.1136/BMJOPEN-2020-047717 34321302 10.1136/bmjopen-2020-047717 PMC8319983

[11] Page MJ, McKenzie JE, Bossuyt PM, Boutron I, Hoffmann TC, Mulrow CD, Shamseer L, Tetzlaff JM, Akl EA, Brennan SE et al (2021) The PRISMA 2020 statement: an updated guideline for reporting systematic reviews. BMJ. 10.1136/BMJ.N71 33782057 10.1136/bmj.n71 PMC8005924

[12] Cumpston M, Li T, Page MJ, Chandler J, Welch VA, Higgins JP, Thomas J (2019) Updated guidance for trusted systematic reviews: a new edition of the Cochrane handbook for systematic reviews of interventions. Cochrane Database Syst Rev 10:ED000142. 10.1002/14651858.ED000142 31643080 10.1002/14651858.ED000142 PMC10284251

[13] Haddaway NR, Grainger MJ, Gray CT (2022) Citationchaser: a tool for transparent and efficient forward and backward citation chasing in systematic searching. Res Synth Methods 13:533–545. 10.1002/JRSM.1563 35472127 10.1002/jrsm.1563

[14] Ouzzani M, Hammady H, Fedorowicz Z, Elmagarmid A (2016) Rayyan-a web and mobile app for systematic reviews. Syst Rev. 10.1186/S13643-016-0384-4 27919275 10.1186/s13643-016-0384-4 PMC5139140

[15] Sterne JAC, Savović J, Page MJ, Elbers RG, Blencowe NS, Boutron I, Cates CJ, Cheng HY, Corbett MS, Eldridge SM et al (2019) RoB 2: a revised tool for assessing risk of bias in randomised trials. BMJ. 10.1136/BMJ.L4898 31462531 10.1136/bmj.l4898

[16] Sterne JA, Hernán MA, Reeves BC, Savović J, Berkman ND, Viswanathan M, Henry D, Altman DG, Ansari MT, Boutron I et al (2016) ROBINS-I: a tool for assessing risk of bias in non-randomised studies of interventions. BMJ. 10.1136/BMJ.I4919 27733354 10.1136/bmj.i4919 PMC5062054

[17] Guyatt G, Oxman AD, Akl EA, Kunz R, Vist G, Brozek J, Norris S, Falck-Ytter Y, Glasziou P, Debeer H (2011) GRADE guidelines: 1. Introduction-GRADE evidence profiles and summary of findings tables. J Clin Epidemiol 64:383–394. 10.1016/J.JCLINEPI.2010.04.026 21195583 10.1016/j.jclinepi.2010.04.026

[18] Higgins JPT, Thompson SG (2002) Quantifying heterogeneity in a meta-analysis. Stat Med 21:1539–1558. 10.1002/SIM.1186 12111919 10.1002/sim.1186

[19] Johnson JR, Burnell-Nugent M, Lossignol D, Ganae-Motan ED, Potts R, Fallon MT (2010) Multicenter, double-blind, randomized, placebo-controlled, parallel-group study of the efficacy, safety, and tolerability of THC:CBD extract and THC extract in patients with intractable cancer-related pain. J Pain Symptom Manage 39:167–179. 10.1016/j.jpainsymman.2009.06.008 19896326 10.1016/j.jpainsymman.2009.06.008

[20] Portenoy RK, Ganae-Motan ED, Allende S, Yanagihara R, Shaiova L, Weinstein S, McQuade R, Wright S, Fallon MT (2012) Nabiximols for opioid-treated cancer patients with poorly-controlled chronic pain: a randomized, placebo-controlled, graded-dose trial. J Pain 13:438–449. 10.1016/j.jpain.2012.01.003 22483680 10.1016/j.jpain.2012.01.003

[21] Fallon M, Giusti R, Aielli F, Hoskin P, Rolke R, Sharma M, Ripamonti CI (2018) Management of cancer pain in adult patients: ESMO clinical practice guidelines. Ann Oncol 29:iv166–iv191. 10.1093/ANNONC/MDY152 30052758 10.1093/annonc/mdy152

[22] Hardy JR, Greer RM, Pelecanos AM, Huggett GE, Kearney AM, Gurgenci TH, Good PD (2025) Medicinal cannabis for symptom control in advanced cancer: a double-blind, placebo-controlled, randomised clinical trial of 1:1 tetrahydrocannabinol and cannabidiol. Support Care Cancer 33:1. 10.1007/S00520-025-09763-5 10.1007/s00520-025-09763-5 PMC12289739 40705150

[23] Hardy J, Haywood A, Gogna G, Martin J, Yates P, Greer R, Good P (2020) Oral medicinal cannabinoids to relieve symptom burden in the palliative care of patients with advanced cancer: a double-blind, placebo-controlled, randomised clinical trial of efficacy and safety of 1:1 Delta-9-Tetrahydrocannabinol (THC) and Cannabidiol …. Trials 21. 10.1186/S13063-020-04541-6 10.1186/s13063-020-04541-6 PMC7336664 32631447

[24] Zylla DM, Eklund J, Gilmore G, Gavenda A, Guggisberg J, VazquezBenitez G, Pawloski PA, Arneson T, Richter S, Birnbaum Aea et al (2021) A randomized trial of medical cannabis in patients with Stage IV cancers to assess feasibility, dose requirements, impact on pain and opioid use, safety, and overall patient satisfaction. Support Care Cancer 29:7471–7478. 10.1007/S00520-021-06301-X 10.1007/s00520-021-06301-x 34085149

[25] Lichtman AH, Lux EA, McQuade R, Rossetti S, Sanchez R, Sun W, Wright S, Kornyeyeva E, Fallon MT (2018) Results of a double-blind, randomized, placebo-controlled study of Nabiximols oromucosal spray as an adjunctive therapy in advanced cancer patients with chronic uncontrolled pain. J Pain Symptom Manage 55:179–188. 10.1016/j.jpainsymman.2017.09.001 28923526 10.1016/j.jpainsymman.2017.09.001

[26] Clarke S, Butcher BE, McLachlan AJ, Henson JD, Rutolo D, Hall S, Vitetta L (2022) Pilot clinical and pharmacokinetic study of Δ9-Tetrahydrocannabinol (THC)/Cannabidiol (CBD) nanoparticle oro-buccal spray in patients with advanced cancer experiencing uncontrolled pain. PLoS ONE 17:e0270543. 10.1371/JOURNAL.PONE.0270543 36240167 10.1371/journal.pone.0270543 PMC9565400

[27] Good PD, Greer RM, Huggett GE, Hardy JR (2020) An open-label pilot study testing the feasibility of assessing total symptom burden in trials of cannabinoid medications in palliative care. J Palliat Med 23:650–655. 10.1089/JPM.2019.0540 31800354 10.1089/jpm.2019.0540 PMC7232640

[28] Pritchard ER, Dayer L, Belz J, Forseth B, Harrington SE, Painter JT (2020) Effect of cannabis on opioid use in patients with cancer receiving palliative care. J Am Pharm Assoc (2003) 60:244–247. 10.1016/J.JAPH.2019.10.013 31843373 10.1016/j.japh.2019.10.013

[29] Aprikian S, Kasvis P, Vigano ML, Hachem Y, Canac-Marquis M, Vigano A (2024) Medical cannabis is effective for cancer-related pain: Quebec Cannabis Registry results. BMJ Support Palliat Care 13:E1285–E1291. 10.1136/SPCARE-2022-004003 37130724 10.1136/spcare-2022-004003

[30] Bramness JG, Hjellvik V, Stubhaug A, Skurtveit S (2022) Possible opioid-saving effect of cannabis-based medicine using individual-based data from the Norwegian prescription database. Basic Clin Pharmacol Toxicol 130:84–92. 10.1111/BCPT.13660 34559439 10.1111/bcpt.13660

[31] Pawasarat IM, Schultz EM, Frisby JC, Mehta S, Angelo MA, Hardy SS, Kim TWB (2020) The efficacy of medical marijuana in the treatment of cancer-related pain. J Palliat Med 23:809–816. 10.1089/JPM.2019.0374 32101075 10.1089/jpm.2019.0374

[32] Sura KT, Kohman L, Huang D, Pasniciuc SV (2022) Experience with medical marijuana for cancer patients in the palliative setting. Cureus 14. 10.7759/CUREUS.26406 10.7759/cureus.26406 PMC9337788 35915672

[33] Webster EM, Yadav GS, Gysler S, McNamara B, Black J, Tymon-Rosario J, Zeybek B, Han C, Arkfeld CK, Andikyan Vea et al (2020) Prescribed medical cannabis in women with gynecologic malignancies: a single-institution survey-based study. Gynecol Oncol Rep. 10.1016/j.gore.2020.100667 33204797 10.1016/j.gore.2020.100667 PMC7653050

[34] Fallon MT, Albert Lux E, McQuade R, Rossetti S, Sanchez R, Sun W, Wright S, Lichtman AH, Kornyeyeva E (2017) Sativex oromucosal spray as adjunctive therapy in advanced cancer patients with chronic pain unalleviated by optimized opioid therapy: Two double-blind, randomized, placebo-controlled Phase 3 studies. Br J Pain 11:119–133. 10.1177/2049463717710042 28785408 10.1177/2049463717710042 PMC5521351

[35] Le Foll B (2021) Opioid-sparing effects of cannabinoids: Myth or reality? Prog Neuropsychopharmacol Biol Psychiatry 106:110065. 10.1016/J.PNPBP.2020.110065 32828853 10.1016/j.pnpbp.2020.110065

[36] Hayes CJ, Krebs EE, Brown J, Li C, Hudson T, Martin BC (2021) Association between pain intensity and discontinuing opioid therapy or transitioning to intermittent opioid therapy after initial long-term opioid therapy: a retrospective cohort study. J Pain 22:1709–1721. 10.1016/J.JPAIN.2021.05.008 34186177 10.1016/j.jpain.2021.05.008 PMC10068896

[37] Cochrane KM, Williams BA, Fischer JAJ, Samson KLI, Pei LX, Karakochuk CD (2020) Regression to the mean: a statistical phenomenon of worthy consideration in anemia research. Curr Dev Nutr 4:nzaa152. 10.1093/CDN/NZAA152 33154991 10.1093/cdn/nzaa152 PMC7596246

[38] Hoch E, Volkow ND, Friemel CM, Lorenzetti V, Freeman TP, Hall W (2024) Cannabis, cannabinoids and health: a review of evidence on risks and medical benefits. Eur Arch Psychiatry Clin Neurosci 275(2):281–292. 10.1007/S00406-024-01880-2 39299947 10.1007/s00406-024-01880-2 PMC11910417

[39] Geum MJ, Yoo SH, Lee SW, Hong M, Jung EH, Kim YJ, Kang B (2025) Impact of CNS medication burden and drug interactions on delirium in patients with advanced cancer: a multicenter prospective observational study. Sci Rep 15(1):39425-. 10.1038/s41598-025-23079-8 41219300 10.1038/s41598-025-23079-8 PMC12606127

[40] (2018) WHO Guidelines for the Pharmacological and Radiotherapeutic Management of Cancer Pain in Adults and Adolescents. World Health Organisation 30776210

[41] Creangă-Murariu I, Froicu EM, Scripcariu DV, Bacoanu G, Poroch M, Moscalu M, Tarniceriu CC, Alexa-Stratulat T, Poroch V (2025) Timing matters: a systematic review of early versus delayed palliative care in advanced cancer. Cancers (Basel) 17. 10.3390/CANCERS17152598/S1 10.3390/cancers17152598 PMC12346342 40805293

[42] Nielsen S, Picco L, Murnion B, Winters B, Matheson J, Graham M, Campbell G, Parvaresh L, Khor KE, Betz-Stablein Bea et al (2022) Opioid-sparing effect of cannabinoids for analgesia: an updated systematic review and meta-analysis of preclinical and clinical studies. Neuropsychopharmacology 47:1315–1330. 10.1038/S41386-022-01322-4 35459926 10.1038/s41386-022-01322-4 PMC9117273

[43] Gewandter JS, Smith SM, Dworkin RH, Turk DC, Gan TJ, Gilron I, Hertz S, Katz NP, Markman JD, Raja Sea et al (2021) Research approaches for evaluating opioid sparing in clinical trials of acute and chronic pain treatments: initiative on methods, measurement, and pain assessment in clinical trials recommendations. Pain 162:2669. 10.1097/J.PAIN.0000000000002283 33863862 10.1097/j.pain.0000000000002283 PMC8497633

[44] Omokore OA, Quadri IO, Kingdom PJ, Ogbuiyi-Chima IC, Meribole SE, Olayinka TO, Ogunnoiki S, Precious S-O, Olaitan A, Chima-Ogbuiyi N et al (2025) Opioids in Cancer Pain Management: A Double-Edged Sword of Relief and Risk. Niger Med J 66:849. 10.71480/NMJ.V66I5.592 41169846 10.71480/nmj.v66i5.592 PMC12571378

[45] Klepstad P, Fladvad T, Skorpen F, Bjordal K, Caraceni A, Dale O, Davies A, Kloke M, Lundström S, Maltoni Mea et al (2011) Influence from genetic variability on opioid use for cancer pain: a European genetic association study of 2294 cancer pain patients. Pain 152:1139–1145. 10.1016/J.PAIN.2011.01.040 21398039 10.1016/j.pain.2011.01.040

